# Three-dimensional computer-guided implant placement in oligodontia

**DOI:** 10.1186/s40729-017-0090-6

**Published:** 2017-07-08

**Authors:** Marieke A. P. Filius, Joep Kraeima, Arjan Vissink, Krista I. Janssen, Gerry M. Raghoebar, Anita Visser

**Affiliations:** 1 Department of Oral and Maxillofacial Surgery, University of Groningen and University Medical Center Groningen, PO Box 30.001, 9700 RB Groningen, The Netherlands; 2 Department of Orthodontics, University of Groningen and University Medical Center Groningen, PO Box 30.001, 9700 RB Groningen, The Netherlands

**Keywords:** Oligodontia, Dental implants, Computer-guided implant dentistry, Guided surgery

## Abstract

**Background:**

The aim of computer-designed surgical templates is to attain higher precision and accuracy of implant placement, particularly for compromised cases.

**Purpose:**

The purpose of this study is to show the benefit of a full three-dimensional virtual workflow to guide implant placement in oligodontia cases where treatment is challenging due compromised bone quantity and limited interdental spaces.

**Patient and methods:**

A full, digitalized workflow was performed for implant placement in two oligodontia patients. Accuracy was assessed by calculating the coordinates of the entry point (shoulder) and apex (tip) as well as the angular deviation of the planned and actual implants.

**Results:**

Implant placement could be well performed with the developed computer-designed templates in oligodontia. Mean shoulder deviation was 1.41 mm (SD 0.55), mean apical deviation was 1.20 mm (SD 0.54) and mean angular deviation was 5.27° (SD 2.51).

**Conclusion:**

Application of computer-designed surgical templates, as described in this technical advanced article, aid in predictable implant placement in oligodontia where bone quantity is scarce and interdental spaces are limited.

## Introduction

Oligodontia is the congenital absence of six or more permanent teeth, excluding third molars [1]. The need for oral rehabilitation in patients with oligodontia is high as they often suffer from functional and aesthetic problems due to a high number of missing teeth. Implant-based prosthodontics seem to be favourable to improve oral function and aesthetics in oligodontia [2].

Implant treatment in oligodontia is, in general, complex. The available bone volume is often limited for implant placement (e.g. above the mandibular nerve) due to jawbone underdevelopment in the area with the agenetic teeth as well as that the bone volume can be reduced due to physiological resorption of the alveolar process after a deciduous tooth without a successor has been lost. Moreover, the available interdental space and angulation of the neighbouring teeth are often unfavourable for implant placement in oligodontia cases.

Computer-designed surgical templates based on (cone beam) computer tomographic ((CB)CT) images have enabled higher precision and accuracy in implant planning [3]. Although this technique is promising, it has, as yet, not been tested in oligodontia. In this technical advanced article, we show the benefit of a full three-dimensional (3D) virtual workflow to guide implant placement in oligodontia, including an analysis of the accuracy of the actual implant placement in both cases.

## Patient and methods

### Implant planning and placement

#### Pre-implant procedure and 3D planning

**Fig. 1:**
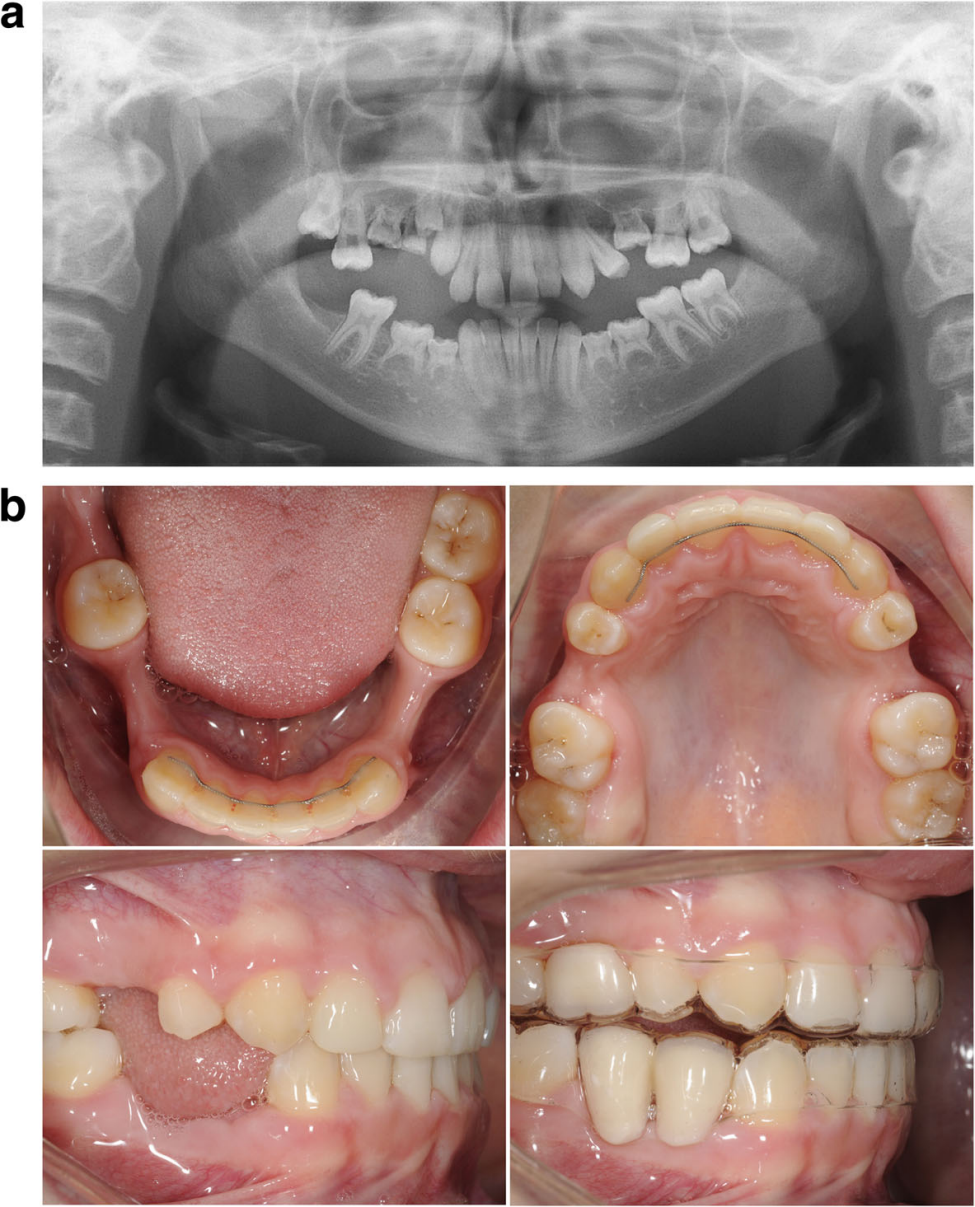
**a** Patient 1—orthopantomogram (OPT) at age of 13. Situation before extraction of the ankylosed deciduous teeth 55, 54, 65, 74, 75, 84, and 85 and start of orthodontic treatment. Eleven permanent teeth (including 4 third molars) were congenitally missing. **b** Patient 1—post-orthodontic situation at age of 16. The top of the mandibular processus alveolaris is small (*upper*). The interdental space at location of the second premolars in the maxilla is 7 and 14 mm at location of the premolars in the mandible. Six dental implants were planned (locations 15, 25, 34, 35, 44 and 45). Implant placement (inclusive bone augmentation with the autogenous retromolar mandibular bone 3 months before implant placement at the place of the 25) was postponed until the age of 18. Essix retainers were used to safeguard the width of the diastemas

**Fig. 2:**
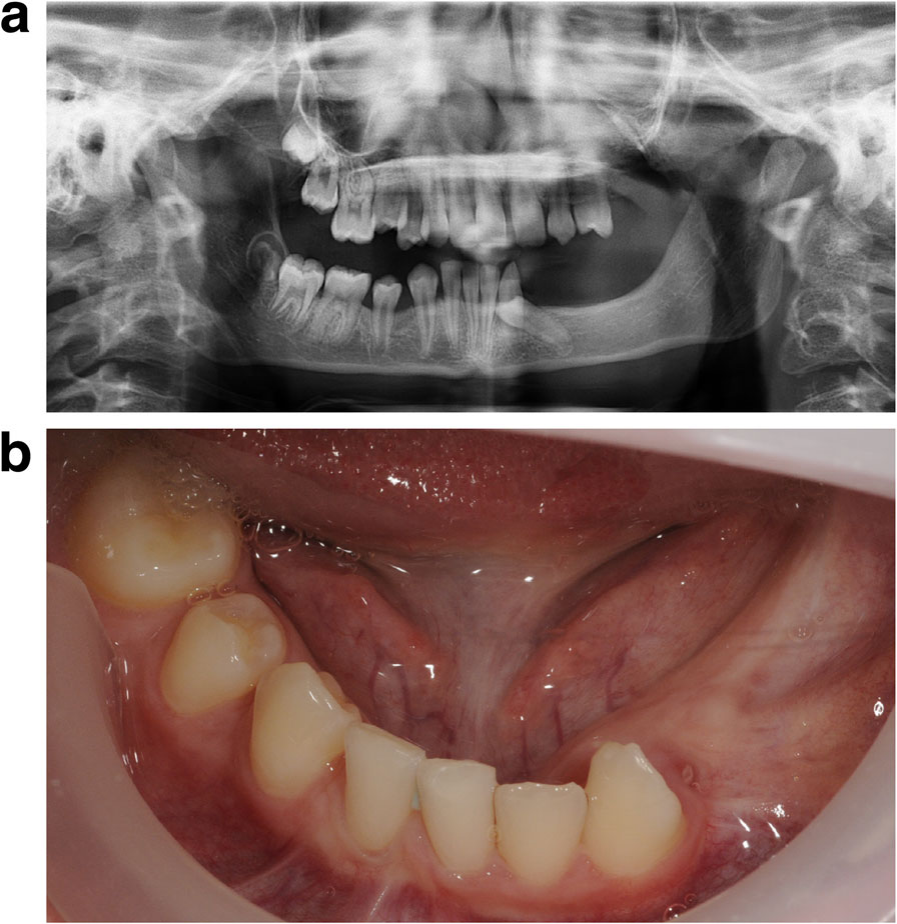
**a** Patient 2—pre-implant orthopantomogram (OPG) at the age of 12. Situation before start of orthodontic and implant treatment. Eleven permanent teeth (including 2 third molars) were congenitally missing and the 34 is impacted. To erect the 34, orthodontic treatment was desired. Due to the lack of stable anchorages in the third quadrant, it was decided to place one implant at tooth region 35 for orthodontic anchorage and future prosthetics. Due to very limited bone height virtual implant planning was needed to avoid damage to the mandibular nerve. **b** Patient 2—mandible, pre implant intra-oral situation at the age of 12. The 34 is not visible in the oral cavity

A CBCT (ICat, Image Sciences International, Hatfield, UK; 576 slices, voxel size 0.3 mm, FOV: 11 × 16 cm) was made of two oligodontia patients (for patient details, see Figs. 1 and 2) for implant planning. Detailed patient information was obtained with regard to the nerve position and bone quality and quantity. In addition, a digital intra-oral scan was made to get a detailed 3D image of the dentition (Chairside Oral Scanner: C.O.S., Lava™).

**Fig. 3:**
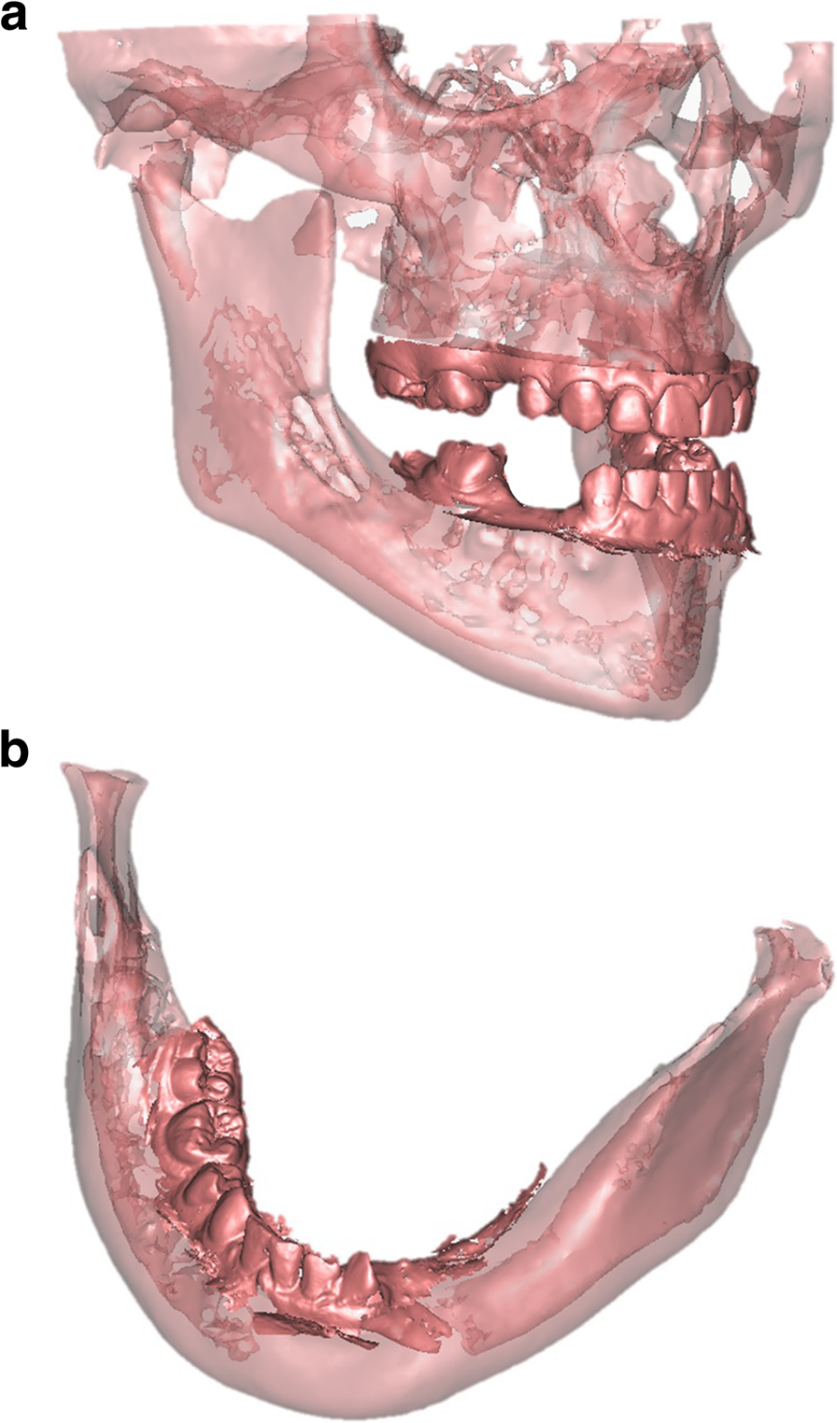
**a** Patient 1—detailed 3D model of the combined data from the CBCT and intra-oral scan at age of 18. **b** Patient 2—detailed 3D model of the combined data from the CBCT and intra-oral scan at age of 12

CBCT and intra-oral scanning data were combined using Simplant Pro (Dentsply, Hasselt, Belgium) in order to obtain a detailed 3D model of both patients (Fig. 3a, b) for virtual implant planning. The intra-oral scans, representing the dentition, were superimposed by a registration process, based on the contour of the corresponding dentition, onto the CBCTs. The intra-oral scan data was imported into the 3D virtual plan software as a *stl*-file. First, the objects representing the upper and lower dentition were globally positioned on the 3D data of the CBCT using manual translation functions. Next, exact positioning was determined using translation and rotation functions, starting in the mid-sagittal plane based on the contour of the model projected on the two-dimensional (2D) CT data. Refinements to the position were made while scrolling through the 2D CBCT data.

**Fig. 4:**
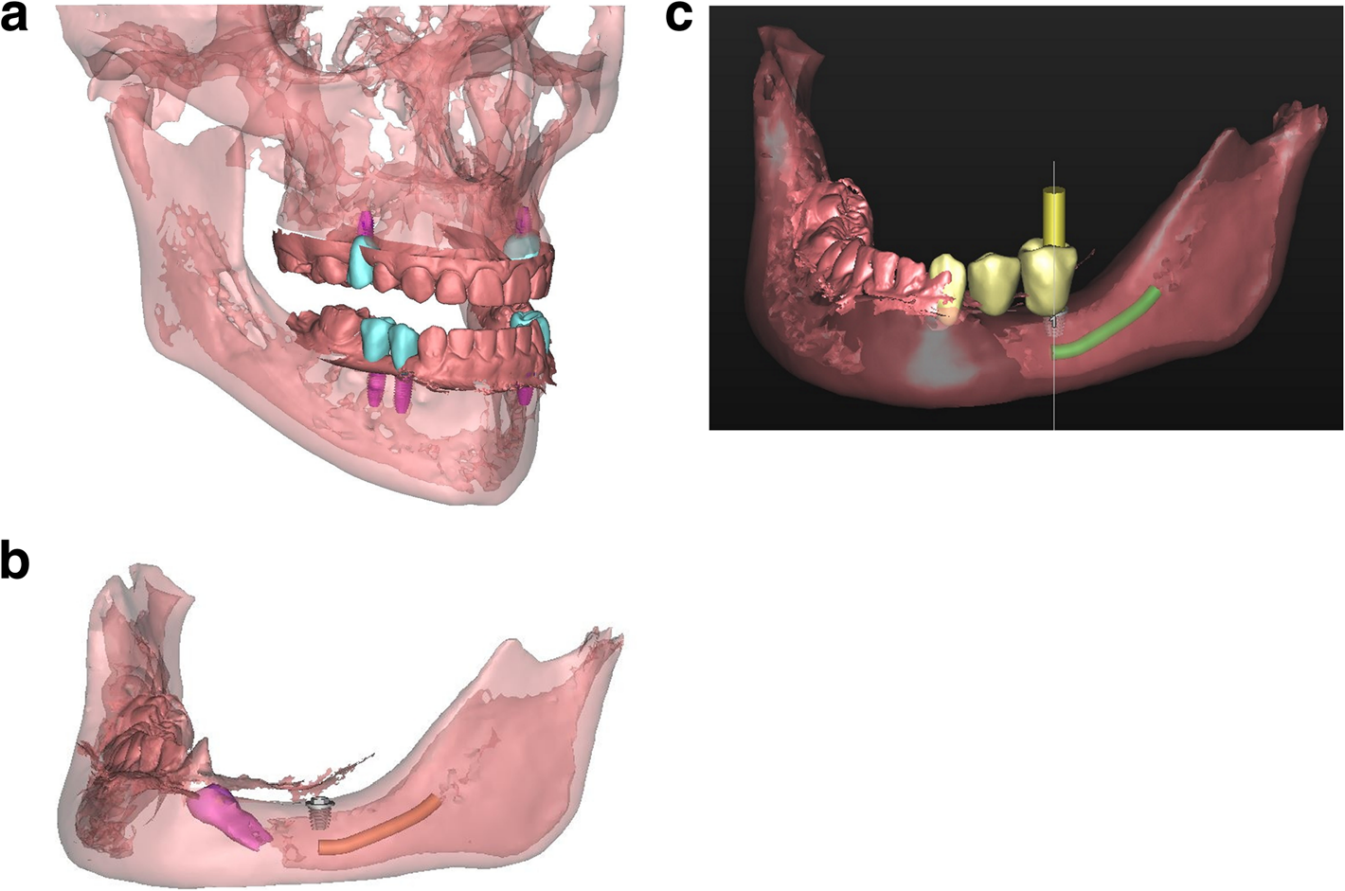
**a** Patient 1—virtual set-up of the ultimate treatment goal. **b** Patient 2—virtual set-up of the ultimate implant position. One short dental implant was planned in region 35, based on the location of the mandibular nerve (*orange*), the impacted 34 (*pink*) and the bone quality and volume. **c** Patient 2—virtual set-up of the ultimate prosthetic treatment goal

Virtual set-ups of the ultimate treatment goal were made for both patients with the virtual planning software Simplant Pro (Fig. 4a–c). Virtual teeth were aligned in the 3D virtual model. Based on the position of these teeth, the implants were planned in the optimal prosthodontic position; tooth size, optimal implant position, location of the mandibular nerve, bone quality and volume and antagonists were also accounted for. The planning was done by the technical physician (J.K.) for both cases, and the implant positions were checked and optimized by the prosthodontist (M.F. and A.V.), orthodontist (K.J.) and surgeon (G.R.).

#### Fabricating 3D templates

**Fig. 5:**
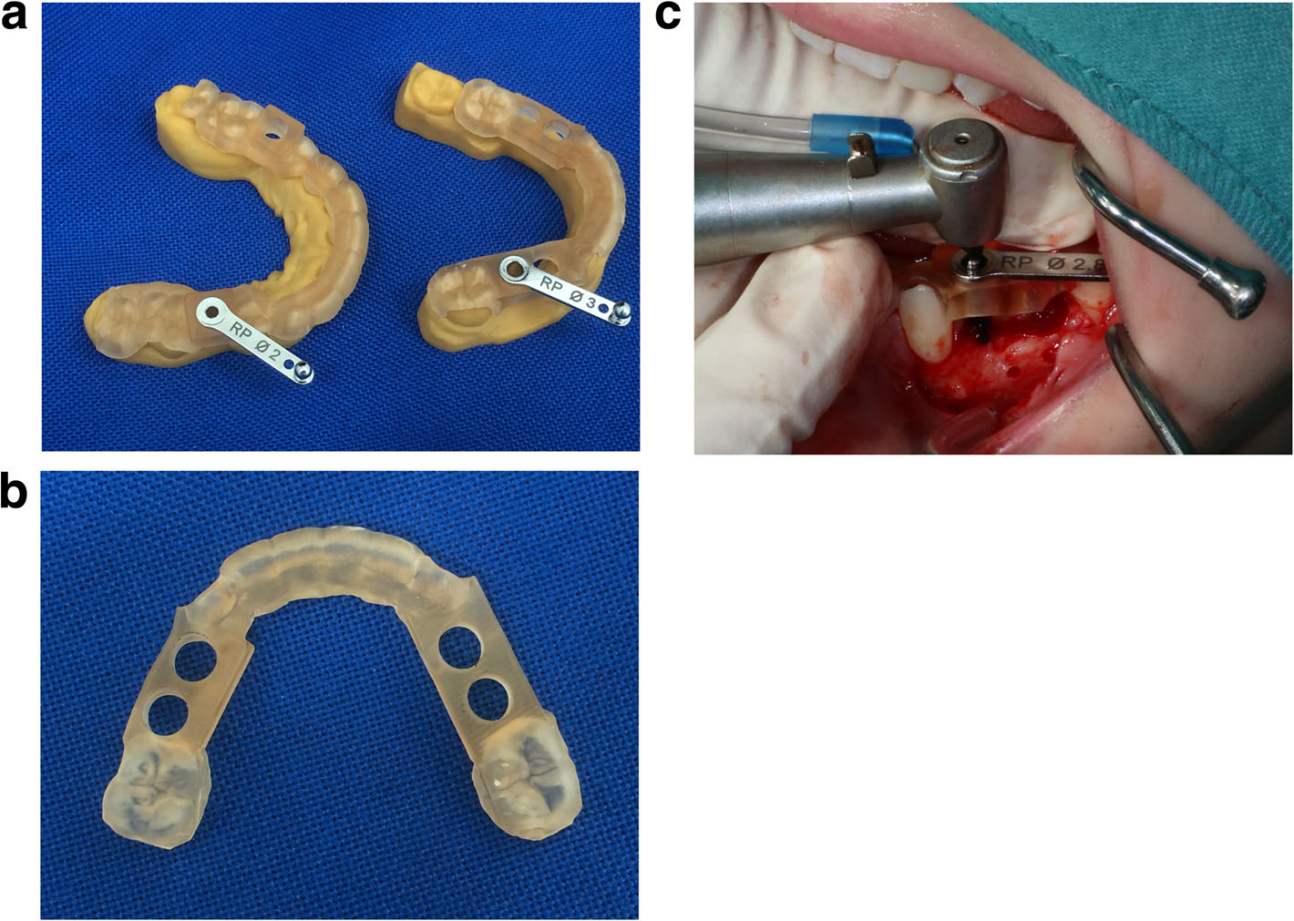
**a** Drilling templates of patient 1. Printed model of the maxilla (*left*) and mandible *(right)* with drilling template and metal drilling inserts (Nobel biocare). **b** Drilling template for the mandible of patient 1. **c** Implant placement of patient 1. Dental implant placement in the mandible using the virtual developed tooth-supported templates and metal drilling inserts

Tooth-supported implant drilling templates were designed by the dental technician, based on the final virtual set-ups using the Geomagic Freeform software (3D Systems, Rock Hill, USA), and then fabricated out of polymethacrylate (Fig. 5a, b). The positioning of each implant was enabled with a 5-mm outer diameter metal drill sleeve (Nobel Guide, Nobel Biocare Holding AG, Zürich-Flughafen, Switzerland; Fig. 5a) as drill sleeves minimize deviation in drill position. The templates were checked for fit and stability in the intra-oral situation.

#### Implant placement

After raising a mucoperiostal flap, the dental implants were placed using the virtual developed tooth-supported drilling templates using metal inserts (Fig. 5c). It was checked whether no dehiscences of the implant surface were present.

## Results

### Clinical and radiographic assessments

The surgical guides fitted well and facilitated implant placement. All implants were placed in the native bone. No dehiscences of the implant surface occurred.

**Fig. 6:**
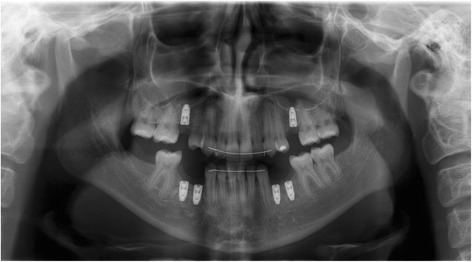
Patient 1—post-operative orthopantomogram (OPT) at age of 18

**Fig. 7:**
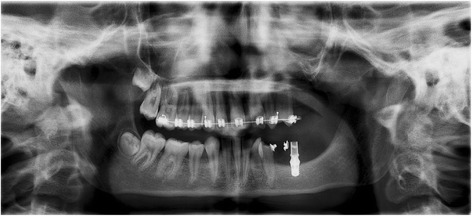
Patient 2—post-operative orthopantomogram (OPT) at age of 13. Situation 10 months after implant placement. Three months after starting the orthodontic treatment, the 34 is already erected

**Fig. 8:**
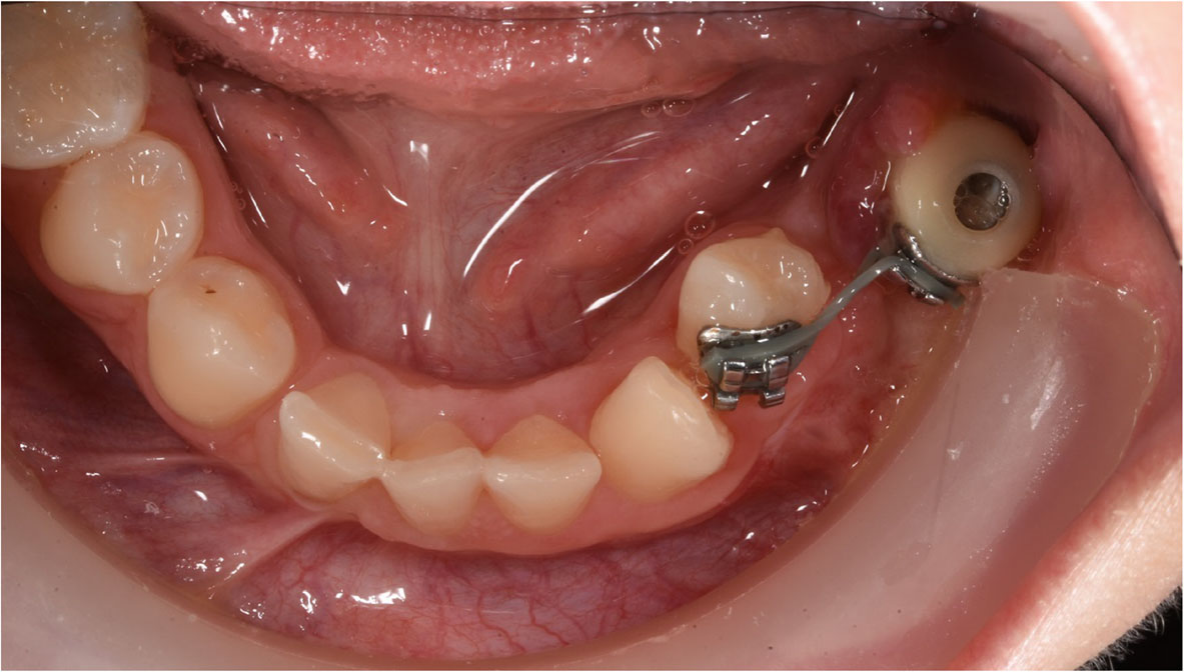
Patient 2—intra-oral situation during orthodontic treatment at the age of 14. A temporary crown with bracket is fixed on the dental implant. Eight months after start of orthodontic treatment, the 34 is already close to the planned end position

**Fig. 9:**
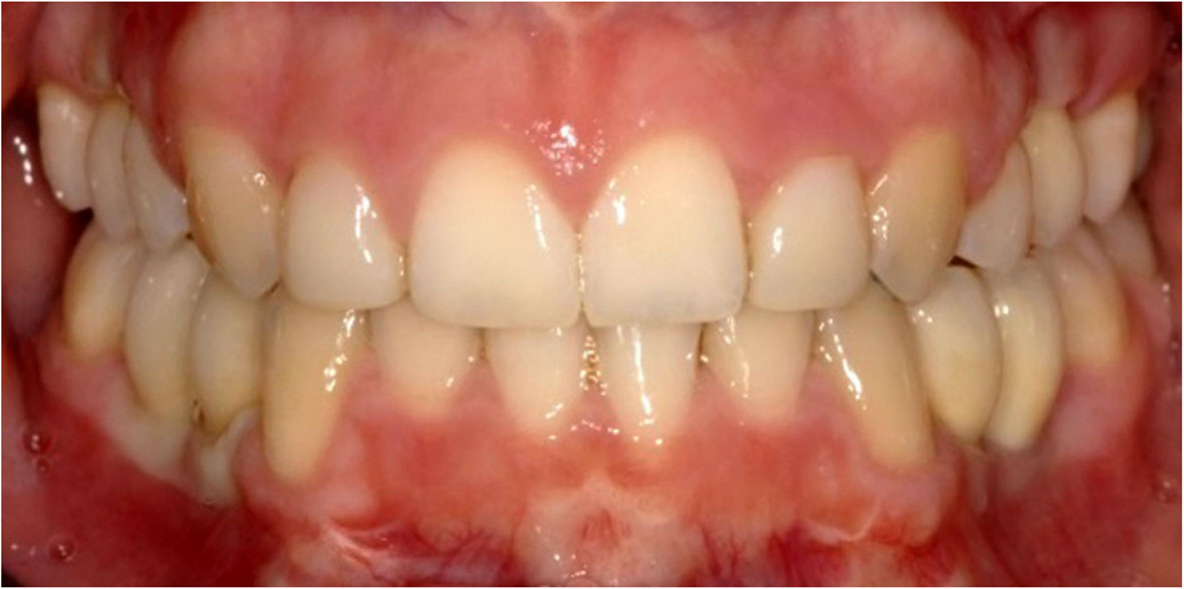
Patient 1—prosthodontic end result 5 months after implant placement

Post-operative orthopantomograms (OPT) of patients 1 and 2 are shown in Figs. 6 and 7. In patient 1, six implants were placed (NobelParallel Conical Connection implants, Nobel Biocare Holding AG, Zürich-Flughafen, Switzerland; Length 8.5 mm; diameter 3.25 mm). In patient 2, one implant (Straumann Standard Plus, Institut Straumann AG, Basel, Switzerland; Length 4.0 mm; diameter 4.1 mm) was placed at region 35. For patient 2, after osseointegration, the temporary prosthetic construction with a bracket to erupt the 34 was placed. Eruption of the 34 was already seen after 3 months of orthodontic treatment (Figs. 7 and 8). Figure 9 shows the prosthodontic end result of patient 1.

### Assessment of accuracy of implant placement

**Fig. 10:**
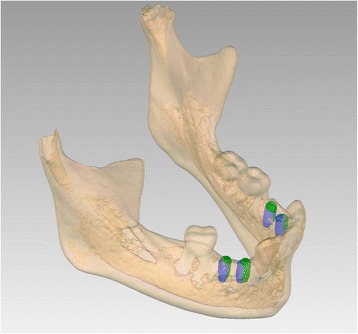
Patient 1—post-operative evaluation of placement accuracy of the implants in the mandible. *Green* is the planned position; *blue* is the actual position

To assess the accuracy of the implant placement, post-operative CBCTs were made of both patients. 3D models of the postoperative result were obtained and superimposed on the data of the implant planning using a surface based alignment method (iterative closest point algorithm) and the same threshold value as used for the pre-operative scans. To deal with the scattering on the post-operative CBCT images in the implant regions, all implants were virtually matched with cylindrical shapes, positioned on the 2D CT data. These cylinders had the same dimensions as the implants and thus adequately represented the implants. The implant placement accuracy was calculated by comparing the pre- and post-implant placement coordinates of the entry point (shoulder), apex (tip) and angular deviation of the implants. Table 1 shows the accuracy data as Euclidian distances (ED) in millimetres (mm) of the entry point (shoulder) and apex (tip) of the implants as well as the degree of angular deviation of all implants (*n* = 7). Mean shoulder deviation was 1.41 mm (SD 0.55); mean apical deviation, 1.20 mm (SD 0.54); and mean angular deviation, 5.27° (SD 2.51). Figure 10 shows the actual differences in the planned and actual location of the implants of patient 1.

**Table 1:** Accuracy data: Euclidian distances (ED, mm) of the apex (tip) and entry point (shoulder) and the degree (°) of angular deviation (axis) of the implants (*n* = 7)

Patient	Location implant (tooth nr)	Shoulder				Tip				Axis			
		*X*	*Y*	*Z*	ED (mm)	*X*	*Y*	*Z*	ED (mm)	*X*	*Y*	*Z*	(°)
1	15 planned	51.52	51.16	47.69		51.69	51.31	56.48		−0.02	−0.02	1.00	
	15 actual	52.85	51.82	48.89	1.91	52.57	50.64	57.60	1.58	0.03	0.13	−0.99	9.10
1	25 planned	90.72	51.04	48.83		90.43	49.74	57.52		0.03	0.15	−0.99	
	25 actual	91.42	50.29	51.04	2.43	90.88	49.60	59.78	2.31	0.06	0.08	−1.00	4.40
1	34 planned	89.21	47.78	16.06		88.12	45.53	24.49		0.12	0.26	−0.96	
	34 actual	88.39	47.86	16.15	0.82	87.90	45.96	24.57	0.49	0.06	0.22	−0.97	4.40
1	35 planned	91.44	51.17	27.24		91.90	53.25	18.72		0.05	0.24	−0.97	
	35 actual	91.02	51.17	26.14	1.18	91.44	54.07	18.00	1.18	−0.05	−0.34	−0.94	5.90
1	44 planned	58.46	48.01	14.10		58.28	46.58	22.77		0.02	0.16	−0.99	
	44 actual	58.34	49.51	13.77	1.54	57.88	46.98	22.02	0.94	0.05	0.29	−0.96	7.90
1	45 planned	55.98	54.54	15.58		55.52	52.54	24.13		0.05	0.23	−0.97	
	45 actual	55.44	54.48	15.03	0.78	54.95	52.78	23.41	0.95	0.06	0.24	−0.97	0.68
2	35 planned	129.71	50.02	66.16		129.68	50.06	71.34		0.01	−0.01	−1.00	
	35 actual	128.5	50.28	66.15	1.24	128.70	49.99	71.31	0.98	−0.04	0.06	−1.00	4.50
Mean					1.41				1.20				5.27
SD					0.55				0.54				2.54

## Discussion

This technical advanced article illustrated the benefit of a full three-dimensional virtual workflow to guide implant placement in oligodontia cases as well as that implants can be reliably placed at the planned positions with the technique proposed.

The described full three-dimensional virtual workflow has several advantages. First, the surgeon is pre-operatively better informed about the requirements for the prosthodontic treatment with regard to the implant position. Second, the patient is pre-operatively better informed about the surgical procedure as well as the prosthodontic end result. The current costs are a limitation of this technique as fully digital planning is more expensive in comparison to a conventional approach. The expectation is that these costs will decrease with the time as this technique will be used more often in the future and probably the costs of the dental technician can also be reduced. At the moment, the extra costs for a full digital planning are reimbursed by Dutch health insurance companies. However, to the best of our knowledge, this (extra) reimbursement is not common in many other countries.

The difference in position between the virtually planned and actually placed implants, according to our workflow, resembles the deviation in implant placement for virtually planned and placed implants in non-oligodontia patients [3–6]. Schneider et al. [4] report in their systematic review a mean deviation of 1.07 mm (95% CI 0.76–1.22 mm) at the shoulder and 1.63 mm (95% CI 1.26–2 mm) at the apex as well as a mean angular deviation of 5.26° (95% CI 3.94–6.58°). More recent studies report similar results [3, 6]. Thus, the accuracy of virtual implant planning in oligodontia patients is comparable to that reported in non-oligodontia cases.

A variety of factors (i.e. technical, product, mechanical, procedure and environmental factors) can affect the accuracy of implant placement [7]. Commonly, implant placement accuracy is higher by experienced surgeons [8], but patient-related factors are often less easy to control. Some progress has been made to control patient factors by using tooth-supported drilling templates, as demonstrated here; they enable a more precise transfer of the virtual implant planning to the surgical site than mucosa- or bone-supported templates [6, 9]. However, there is still a need to identify appropriate evaluation techniques and mechanisms capable of optimizing transfer precision and eliminating errors of three-dimensional planning and guiding systems for the partially dentate jaw [10]. Planning is complex, and high transfer precision is not always easy to accomplish, particularly in oligodontia cases with a large number of missing teeth. With the use of the described method, pre-operative implant planning is possible and placement is more predictable.

## Conclusion

This technical advanced article introduces a fully digitalized workflow for implant planning in complex oligodontia cases. The application of computer-designed surgical templates enables predictable implant placement in oligodontia, where bone quantity and limited interdental spaces can be challenging for implant placement. The stepwise approach described in this technical advanced article provides the dentist and surgeon with a basis to plan and guide the preferred implant placement in oligodontia cases.

## Abbreviations

(CB)CT: (Cone beam) computer tomography
2D: Two-dimensional
3D: Three-dimensional
ED: Euclidian distances
OPT: Orthopantomogram
